# At-risk but viable myocardium in a large animal model of non ST-segment elevation acute coronary syndrome: cardiovascular magnetic resonance with *ex vivo* validation

**DOI:** 10.1186/1532-429X-15-94

**Published:** 2013-10-09

**Authors:** Henry Chang, Tam Tran, George E Billman, Mark W Julian, Robert L Hamlin, Orlando P Simonetti, Giuseppe Ambrosio, Peter B Baker, Guohong Shao, Elliott D Crouser, Subha V Raman

**Affiliations:** 1 Dorothy M. Davis Heart and Lung Research Institute, The Ohio State University, 473 W 12th Ave, Suite 200, Columbus, OH 43210, USA; 2 Department of Physiology and Cell Biology, OSU, 370 W 9th Ave, Columbus, OH 43210, USA; 3 Department of Veterinary Biosciences, OSU, 1900 Coffey Road, Columbus, OH 43210, USA; 4 Division of Cardiovascular Medicine, OSU, 473 W 12th Ave, Columbus, OH 43210, USA; 5 Department of Radiology, OSU, 395 W 12th Ave, Columbus, OH 43210, USA; 6 Division of Cardiology, University of Perugia, Ospedale S. Maria della Misericordia, Via S. Andrea delle fratte, 06156 Perugia, Italy; 7 Department of Pathology, OSU and Nationwide Children’s Hospital, 700 Children’s Dr, Columbus, OH 43205, USA; 8 Division of Pulmonary, Allergy, Critical Care & Sleep Medicine, OSU, 473 W 12th Ave, Columbus, OH 43210, USA

**Keywords:** Myocardial ischemia, Oxygen consumption, Cardiovascular magnetic resonance, Canine model, Mitochondria

## Abstract

**Background:**

Patients with non-ST-segment elevation acute coronary syndrome (NSTE-ACS) have varying degrees of salvageable myocardium at risk of irreversible injury. We hypothesized that a novel model of NSTE-ACS produces acute myocardial injury, measured by increased T2 cardiovascular magnetic resonance (CMR), without significant necrosis by late gadolinium enhancement (LGE).

**Methods:**

In a canine model, partial coronary stenosis was created and electrodes placed on the epicardium. Myocardial T2, an indicator of at-risk myocardium, was measured pre- and post-tachycardic pacing.

**Results:**

Serum troponin-I (TnI) was not detectable in unoperated sham animals but averaged 1.97 ± 0.72 ng/mL in model animals. Coronary stenosis and pacing produced significantly higher T2 in the affected vs. the remote myocardium (53.2 ± 4.9 vs. 43.6 ± 2.8 ms, p < 0.01) with no evident injury by LGE. Microscopy revealed no significant irreversible cellular injury. Relative respiration rate (RRR) of affected vs. remote myocardial tissue was significantly lower in model vs. sham animals (0.72 ± 0.07 vs. 1.04 ± 0.07, p < 0.001). Lower RRR corresponded to higher final TnI levels (R^2^ = 0.83, p = 0.004) and changes in *CaMKIID* and mitochondrial gene expression.

**Conclusions:**

A large animal NSTE-ACS model with mild TnI elevation and without ST elevation, similar to the human syndrome, demonstrates signs of acute myocardial injury by T2-CMR without significant irreversible damage. Reduced tissue respiration and associated adaptations of critical metabolic pathways correspond to increased myocardial injury by serum biomarkers in this model. T2-CMR as a biomarker of at-risk but salvageable myocardium warrants further consideration in preclinical and clinical studies of NSTE-ACS.

## Background

Non ST-segment elevation acute coronary syndrome (NSTE-ACS) – a subset of acute coronary syndrome (ACS) that includes non ST-segment elevation myocardial infarction (NSTEMI) and unstable angina (UA) – is responsible for nearly 1 million emergency department presentations each year in the United States alone [1,2]. While patients with ST-elevation myocardial infarction (STEMI) have well defined evaluation and management pathways, NSTE-ACS patients receive heterogeneous care yet suffer similar if not greater morbidity and mortality [3]. Paradoxically, there is worse adherence to guideline-based management in higher risk vs. lower risk NSTE-ACS patients [4]. At least some of the heterogeneity in initial management likely stems from uncertainty regarding which patients harbor at-risk but not yet irreversibly injured myocardium that might benefit from accelerated invasive care.

While serum biomarkers of myocardial necrosis reliably indicate that damage has occurred, they cannot identify myocardium that is at-risk but still salvageable. Cardiovascular magnetic resonance (CMR) with myocardial T2 imaging has shown value as a noninvasive imaging biomarker of at-risk myocardium, delineating myocardium at-risk in both a large animal model of STEMI [5] as well as in patients with STEMI [6]. Our group has previously shown that CMR with T2 imaging may identify at-risk but salvageable myocardium in patients with NSTE-ACS [7], prompting us to seek further mechanistic understanding for the basis of T2 increase in this syndrome. In the present study, we tested the hypotheses that i) T2 elevation without late gadolinium enhancement (LGE) is present in a large animal model of NSTE-ACS and ii) this finding represents acutely injured myocardium that remains salvageable by *ex vivo* tissue analyses, despite being at risk for subsequent irreversible damage, as reflected by the induction of cardioprotective metabolic adaptations characteristic of ischemia-induced myocardial hibernation [8,9].

## Methods

### Animal preparation

**Figure 1:**
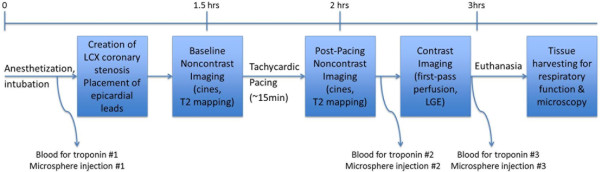
**Experiment procedure timeline.** The diagram provides an overview of experimental procedures for the NSTE-ACS model. Sham animals underwent neither stenosis creation nor pacing, and 2 control animals had no thoracotomy.

An overview of the study protocol is shown in Figure 1. All the animal procedures were approved by The Ohio State University Institutional Animal Care and Use Committee and conformed to the *Guide for the Care and Use of Laboratory Animals* published by the US National Institutes of Health. Thirteen heartworm-free, random-source, mixed-breed dogs (aged 1-3 years, weight 11.4-15.9 kg) were anesthetized using ketamine (5-8 mg/kg, IM) and inhaled isoflurane (1-2% in 95% O_2_) and remained anesthetized throughout all experimental procedures. In 2 randomized dogs, no surgical operations were performed (unoperated shams). ECG was monitored via surface electrodes. After left thoracotomy in the remaining 11 dogs, a catheter was placed in the left atrial appendage for microsphere injection. In a random selection of 4 dogs, no further operations were performed prior to closing of the chest wall (shams). In the 7 model dogs, an 18-gauge needle was laid flat on the left circumflex coronary artery (LCx) then removed once a suture was secured around the artery and needle, leaving a lumen constricted to the nominal outer diameter of the needle (~1.27 mm). Copper pacing wires were then threaded through the epicardium of the right ventricle and connected to an external electrical stimulator for later myocardial pacing. Standard chest wall closing procedures and post-operative anesthesia and analgesia protocols were then followed [10].

Serum isolated from peripheral blood drawn at 3 time points (0-baseline prior to surgery, 1-post-stenosis and post-pacing in model dogs or at the end of CMR examination in sham dogs, and 2-just before euthanasia) was examined for troponin I (TnI) content using a high-sensitivity assay according to the manufacturer’s recommendations (TnI-Ultra assay and ADVIA Centaur XP immunoanalyzer, Siemens Healthcare Diagnostics; Malvern, PA).

Based on the anatomical perfusion territory of the LCx and preliminary results from pilot studies, the lateral LV myocardium at the level of the posterior papillary muscle was denoted as the affected region while a section of the anterior LV wall near the anterior papillary muscle was designated as the remote region. At the conclusion of each imaging experiment, the animal was euthanized and the heart immediately harvested. Myocardium from affected and remote regions was excised using the papillary muscles as anatomical landmarks. Tissue samples from each myocardial region were obtained for immediate tissue respiration, wet-to-dry weight and tissue necrosis analyses on fresh tissue. Additional samples were acquired and either fixed or frozen (-80°C) for later light and electron microscopy or gene expression analyses, respectively, as discussed below and in the provided online supplement (see Additional file 1).

### CMR protocol

CMR was initiated within 30 minutes of chest wall closure. All CMR scans were performed on a 1.5 Tesla system with a 12-element phased-array cardiac coil (MAGNETOM Avanto, Siemens Medical Solutions, Inc.; Erlangen, Germany). Identical imaging protocols were used in both groups of animals, NSTE-ACS model and sham. In the NSTE-ACS model dogs, scans were performed immediately after 15 minutes of epicardial pacing at 180-210 beats/min (60-70% of normal canine maximum heart rate vs. average stable post-anesthesia heart rate of 100 beats/min). We acquired multiplane cines to measure LV systolic function, T2 maps to identify areas of acute injury, first-pass perfusion imaging of microcirculatory myocardial blood flow, and LGE images to visualize areas of necrosis. Further detailed information is provided in Additional file 1.

### Image analysis

Two investigators analyzed all images by consensus review (HC and TT). For analysis of T2 maps, myocardial regions of interest (ROIs) were drawn encompassing the affected region and remote region for each dog (typically in the regions of segments 9 and 12 by standardized LV segmentation nomenclature [11]), and the mean T2 in each ROI was recorded. Perfusion images were visually scored by counting the number of LV segments that showed abnormalities. LGE images were similarly evaluated using consistent display settings. LV ejection fraction was calculated using Simpson’s rule and manual segmentation of the endocardial border on end-systolic and end-diastolic frames of contiguous short-axis cines.

### Coronary blood flow determination via microspheres

To verify the changes in coronary blood flow due to stenosis and pacing, we injected fluorescent microspheres (BioPAL, Inc.; Worcester, MA) into the left atrium of 5 animals (2 NSTE-ACS model and 3 sham) at three time points, each with a different fluorescent label: 1) prior to surgical stenosis of the LCx (gold), after stenosis and pacing (samarium), and just prior to euthanasia (lutetium). The disintegrations per minute (dpm) of each label was quantified (BioPAL, Inc.) in tissue sections (~1 gram) obtained from both the affected and remote regions. After calibration, the dpm of each label was converted to flow in the affected region relative to the flow in the remote region at each of the three time points.

### Myocardial tissue necrosis and edema determination

Examinations were made on myocardial tissue sections from both the affected and remote regions in all animals to determine the presence of tissue necrosis and edema using established 2,3,5-triphenyltetrazolium chloride (TTC) staining and wet/dry weight ratio measurement, respectively, as described further in Additional file 1.

### Light and electron microscopy

Tissue sections harvested from the remote and affected myocardial regions were routinely processed and stained [12] (further detailed in Additional file 1). Tissue histology was graded blindly for evidence of structural and/or cellular injury/abnormalities by pathologist (PBB) review. High-resolution electron microscopy digital image analysis of myocardial mitochondrial ultrastructure was performed [13], and values obtained for the affected myocardial mitochondria were normalized to those averaged from the corresponding remote myocardial mitochondria for the sake of comparison between the groups.

### Ex vivo myocardial tissue respiration

To evaluate the effect of ischemia upon cellular respiration in our NSTE-ACS model, O_2_ consumption by myocardial tissue (~0.2-0.3 grams) obtained from both the affected and remote regions was measured in all animals employing standard techniques as further described in Additional file 1. The rate of mitochondrial respiration in the affected region was normalized to that of the remote region in each animal and compared between the model and sham groups.

### Myocardial gene expression

Frozen harvested tissue samples from both the affected and remote myocardial regions obtained from each animal were processed according to standard techniques to isolate high quality total RNA, as detailed in Additional file 1. Gene expression for select canine genes in both the affected and remote myocardial regions was determined using quantitative real-time PCR. Relative copy numbers and expression ratios for all genes of interest were normalized to that of the housekeeping gene, *β-actin*, and then compared within the model group.

### Statistical analysis

Results are reported as mean ± standard deviation (SD). *In vivo* and *ex vivo* measurements for affected myocardial tissue respiration in model vs. sham groups were compared by use of unpaired *t*-tests. Delta T2 (affected minus remote) was also compared between the two groups using unpaired *t*-tests. For serial microsphere and TnI data, a two-way analysis of variance (ANOVA) test was performed to examine the individual contributions of animal group and sampling time, as well as their interaction effects. The T2 data was also analyzed in this way using animal group and region (remote and affected) as the main effects. Comparisons of selective gene expression between the affected and remote regions within the model group were made using the Wilcoxon Rank-Sum test. Statistical significance was based upon a value of *p* < 0.05.

## Results

**Figure 2:**
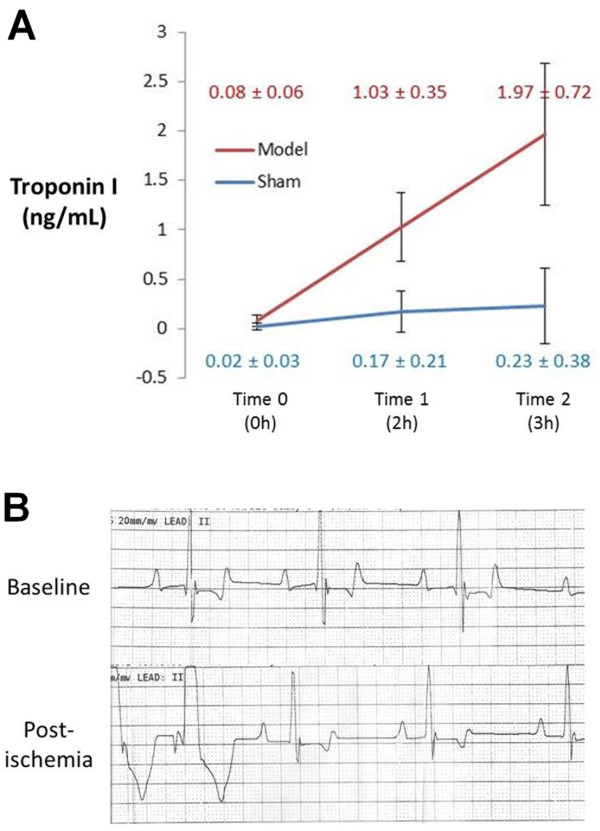
**Troponin I and ECG. A)** Blood was drawn for troponin-I (TnI) measurement at three time points: baseline/pre-surgery (Time 0), post-stenosis and post-pacing in model dogs or at the end of CMR examination in sham dogs (Time 1, approximately 2 hours after surgery), and just before euthanasia (Time 2, approximately 3 hours after surgery). While thoracotomy and instrumentation produced minimal TnI elevation in operated sham animals, TnI was significantly higher (though only mildly elevated) in the NSTE-ACS model. **B)** Representative ECG tracings at baseline and after ischemia (stenosis and pacing) demonstrated premature ventricular contractions but no ST elevation with ischemic conditions in the NSTE-ACS model.

Of the seven dogs in the NSTE-ACS model group, five survived the entire experiment. Two expired due to ventricular fibrillation – one during the initial surgery and the other during pacing. No pre-euthanasia deaths occurred in the sham group. No coronary artery anomalies were identified with the left circumflex artery observed to supply the lateral LV wall in all operated animals. TnI was undetectable on serial measurement in the serum of the 2 unoperated control animals, while thoracotomy and cardiac instrumentation presumably produced the borderline TnI elevation (0.44 ± 0.48 ng/mL at final measurement) in operated sham animals. The NSTE-ACS model resulted in final TnI levels of 1.97 ± 0.72 ng/mL, which was significantly higher than the operated sham animals (p = 0.02) and the averaged final TnI levels across all sham animals (0.27 ± 0.41 ng/mL, *p* < 0.01) (Figure 2A); no significant ST segment elevation was observed in the NSTE-ACS model animals throughout any of the experiments (Figure 2B). Two-way ANOVA showed significant contributions to TnI changes due to time (*p* < 0.001), animal group (*p* < 0.001), and time-group interaction (*p* < 0.001).

### In vivo imaging

**Figure 3:**
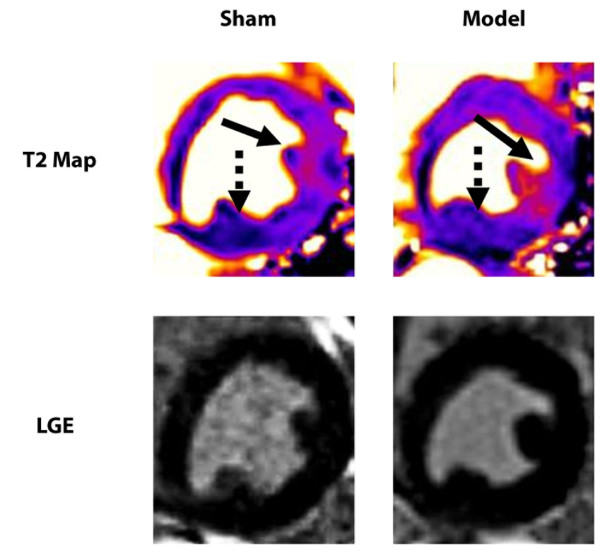
**Myocardium at risk without necrosis.** T2 maps and late gadolinium enhancement (LGE) images are shown in a mid short-axis plane from representative sham and NSTE-ACS model animals. In the sham, mild T2 elevation was observed in the affected area (solid arrow) compared to the remote region (dotted arrow). In the model, creation of left circumflex coronary artery stenosis and tachycardic pacing produced significantly more pronounced myocardial T2 elevation in the affected area. LGE demonstrated no myocardial enhancement.

**Figure 4:**
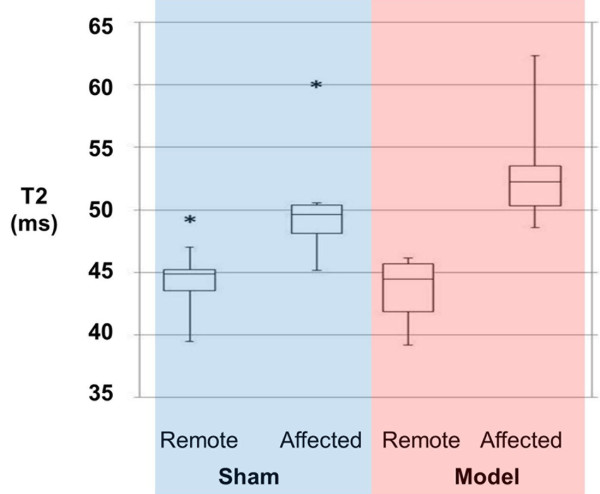
**T2 Values.** T2 values from remote and affected myocardial regions are shown for the sham and the NSTE-ACS model groups. The T2 difference (affected – remote) was significantly larger in the model group than in the sham group (9.6 ± 4.2 ms vs. 5.1 ± 1.2 ms, *p* = 0.02). Asterisks indicate outlier values from one sham dog.

LV ejection fraction by CMR acquired during anesthesia was mildly reduced in the model dogs with LCx stenosis post-pacing compared to the sham dogs (40 ± 2 vs. 48 ± 8%, *p* = 0.03). Myocardial T2 was increased in the LCx-territory myocardium relative to remote myocardium in NSTE-ACS model dogs after pacing (Figure 3), averaging 53.2 ± 4.9 ms in the affected region vs. 43.6 ± 2.8 ms in the remote region. A smaller regional difference in myocardial T2 was also present in sham animals (48.6 ± 2.1 vs. 43.4 ± 2.9 ms; Figure 4), somewhat larger in those that were unoperated (46.3 ± 1.6 vs. 40.0 ± 0.6 ms) vs. operated (49.7 ± 1.1 vs. 45.2 ± 1.4 ms). Two-way ANOVA showed significant contributions to T2 due to myocardial region (*p* < 0.001), animal group (*p* = 0.02), and region-group interaction (*p* = 0.03). Perfusion and LGE images showed no first-pass defect, microvascular obstruction or enhancement (Figure 3) in any model or sham-operated animals.

### Myocardial blood flow and tissue oxygen metabolism

**Figure 5:**
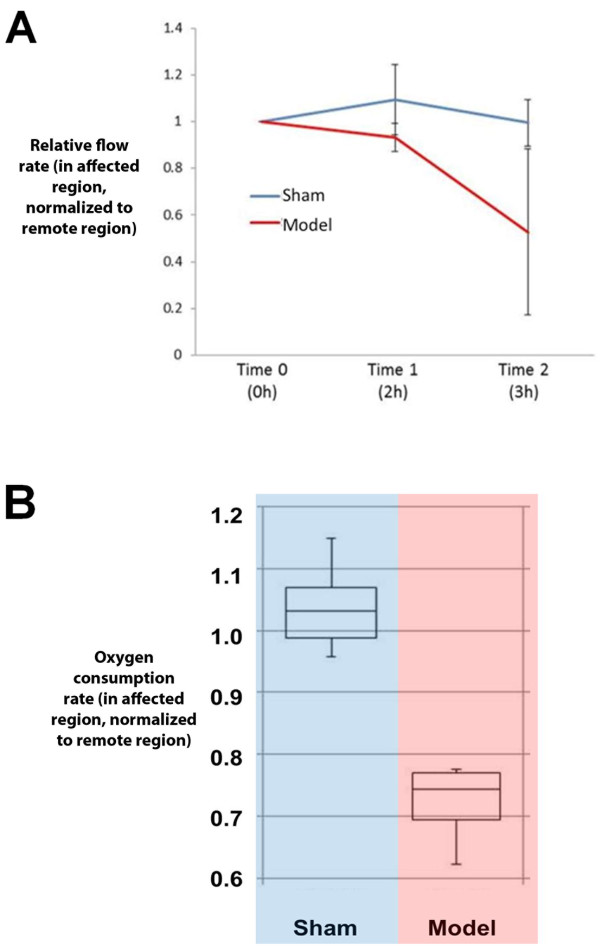
**Myocardial blood flow and respiration. A)** The change in myocardial blood flow through the affected region (relative to the flow through the remote region at that point in time) is shown at three time points: baseline (Time 0), post-stenosis and post-pacing or at the end of imaging in sham animals (Time 1), and just before euthanasia (Time 2), with a significant decrease in blood flow with the model. **B)***Ex vivo* myocardial respiration rates in the affected region normalized to rates in the remote region were significantly lower in the NSTE-ACS model vs. that in sham animals.

An average of 38,395 microspheres was collected for each reading. In the NSTE-ACS model dogs, the flow through the affected region relative to the flow through the remote region decreased after stenosis and pacing with a mean end-experiment flow ratio of 0.53 ± 0.36. Lack of stenosis and pacing in the sham dogs resulted in no change in myocardial blood flow over the course of the experiment where end-experiment flow ratio averaged 1.0 ± 0.10 (Figure 5A). Two-way ANOVA showed significant contributions to this flow ratio due to time (*p* < 0.001), animal group (*p* < 0.001), and time-group interaction (*p* < 0.001). In six sham animals, the myocardial respiration rate from the affected and remote regions was 0.44 ± 0.10 nmol O_2_/mg/min and 0.43 ± 0.08 nmol O_2_/mg/min, respectively, yielding an average respiration rate ratio of 1.04 ± 0.07. In four NSTE-ACS model dogs, the myocardial respiration rate from the affected and remote regions was 0.44 ± 0.07 nmol O_2_/mg/min and 0.61 ± 0.05 nmol O_2_/mg/min, respectively, resulting in a significantly lower respiration rate ratio of 0.72 ± 0.07 when compared to the sham group (*p* < 0.001, Figure 5B). These findings are in keeping with previous investigations wherein tissue hypoxia was shown to limit pacing-induced aerobic respiration [14], indicating that the affected myocardium in the NSTE-ACS model was operating at or near its maximal aerobic capacity at baseline.

### Tissue pathology

Immediate post-mortem analysis of fresh tissue revealed comparable wet-to-dry ratios in the affected region in the NSTE-ACS dogs compared to the matching remote regions or compared to matching myocardial tissues from sham animals. In addition, the results demonstrated no change in wet-to-dry ratios related to the surgical preparation (Additional file 1: Table S3). Staining of fresh tissues obtained from the affected and remote myocardial regions with TTC was negative indicating an absence of non-viable myocardium in tissue samples (2-3 mm slice thickness) corresponding to the abnormal T2 signal by this technique (Additional file 1: Figure S1).

**Figure 6:**
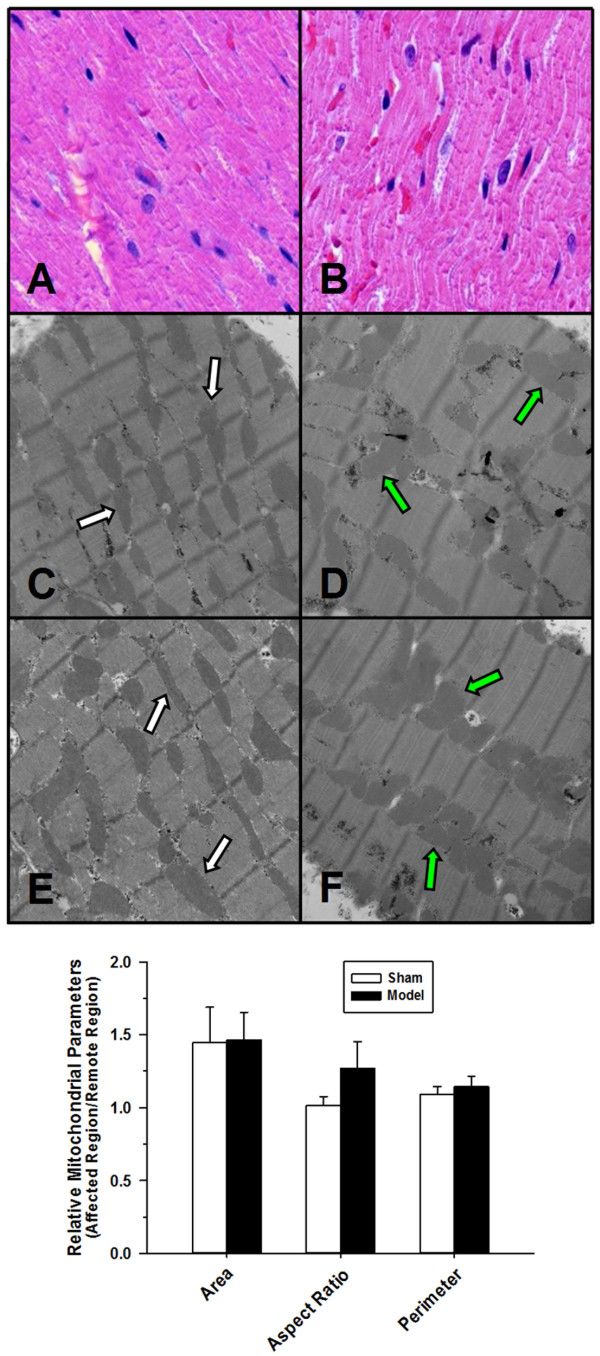
**Microscopy.** Top) Representative light photomicrographs from animals in the sham **(A)** vs. NSTE-ACS model **(B)** groups. H&E staining revealed no significant contraction band necrosis to indicate irreversible cell death in either group. Middle) Representative electron photomicrographs of left ventricular myocardial tissue (noting the mitochondria – arrows) corresponding to the remote region from a representative sham animal **(C**, white**)**, the matching affected region from the same sham animal **(D**, green**)**, the remote region from a representative animal from the model group **(E**, white**)** and the matching affected region from the same model animal **(F**, green**)** (Original magnification: 11,000×; Staining: 2% uranyl acetate and Reynolds lead citrate). Bottom) Electron microscopy showed no evidence of mitochondrial damage (e.g., decreased matrix density, disintegration of cristae) or structural signs of irreversible muscle damage. Although mitochondria were noted to be consistently larger in the lateral LV wall compared to the anterior LV wall in controls and NSTE-ACS model animals, there was no objective evidence of mitochondrial swelling, as reflected by computer-derived measures of mitochondrial transectional area, perimeter and aspect ratio, in the NSTE-ACS group. Data presented as ratios of measurements made on mitochondria within the affected region relative to those made on corresponding mitochondria within the remote region in the same animal.

In keeping with the TTC findings and negative LGE results on CMR, light microscopy generally showed no increase in contraction band necrosis, a marker of irreversible cellular injury, in the affected region. Small areas with contraction band-like patterns were seen in both sham and model groups, suggesting an artifact of the tissue cutting and preparation process or transient myocardial fasciculations in the immediate post-mortem period. Unexpectedly, mitochondrial size was statistically greater in the myocardium of the lateral wall compared to the anterior wall in all animals, but normalized data indicated no mitochondrial swelling relative to controls or other manifestations of mitochondrial damage in the NSTE-ACS dogs (Figure 6).

### CaMKIID and mitochondrial gene expression during NSTE-ACS

**Figure 7:**
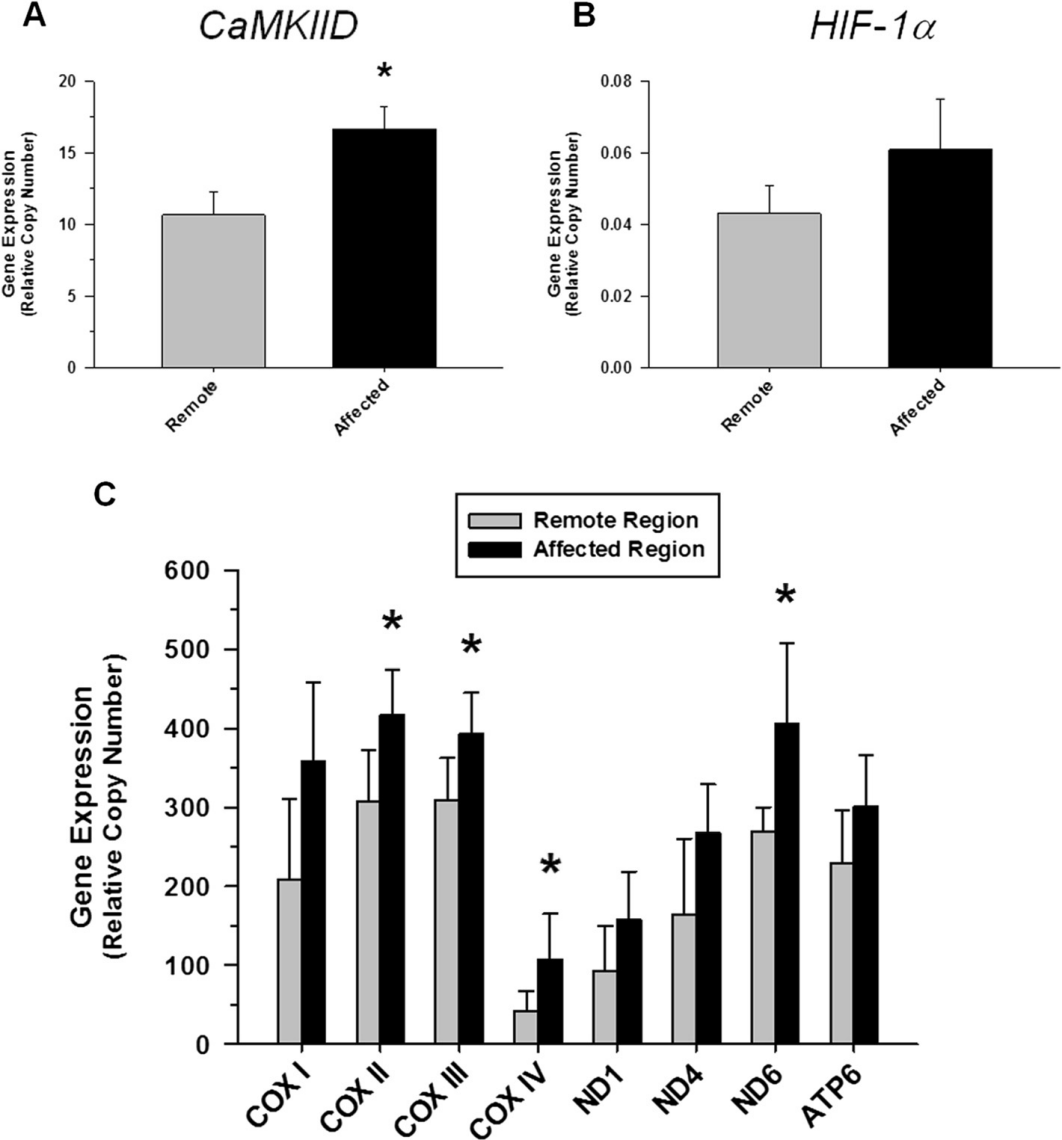
**Gene expression.** Quantitative real-time PCR was used to measure nuclear *CaMKIID***(A)**, *HIF-1α***(B)** and mitochondria- related gene expression **(C)** in the remote and affected myocardial regions in the NSTE-ACS model group. The NSTE-ACS model was associated with significant induction of the expression of *CaMKIID* and multiple mitochondria-related genes representing components of each cytochrome complex in the affected region compared to the remote (non-ischemic) region.

Enhanced expression of *CaMKIID*, a pleiotropic kinase that is responsive to changes in intracellular calcium [15], was observed within 3 hours in the affected myocardium in the NSTE-ACS model (Figure 7A). There was no significant change in *HIF-1α* gene expression in the affected myocardium (Figure 7B). In keeping with previous studies showing that myocardial mitochondrial biogenesis is promoted in response to ischemia [16], T2 changes within the affected myocardium of the NSTE-ACS animals correlated with increased mitochondria-related gene expression (Figure 7C).

## Discussion

There are two important and novel findings of the present study: 1) a canine model based upon reduced myocardial oxygen supply (coronary stenosis) and increased myocardial oxygen demand (tachycardic pacing) mimics key features of clinical NSTE-ACS and 2) increased myocardial T2 conforms to an early form of myocardial damage that is associated with viable myocardium, as indicated by the absence of late gadolinium enhancement on CMR and other signs of irreversible myocardial damage. These *in vivo* imaging findings corresponded to a reduction in tissue respiration without signs of irreversible cell or mitochondrial injury by *ex vivo* analyses. Together, this is in keeping with an adaptive state such as myocardial stunning that defines myocardium at risk for subsequent irreversible injury and adverse remodeling [17]. Indeed, the observed decline in myocardial blood flow by microsphere analysis and increase in myocardial injury by TnI just prior to euthanasia suggests that the earlier identification of myocardium with increased T2 but no LGE identifies at-risk but potentially salvageable myocardium.

NSTE-ACS encompasses a wide range of pathophysiologies, but a clear distinction from STEMI is its relative lack of complete/sustained coronary artery occlusion [18]. Without complete thrombotic occlusion, patients presenting with NSTE-ACS typically lack the obvious ST elevation by ECG and the more dramatic symptoms of patients with STEMI. As a result, they are not treated with the same urgency in the community, emergency department or hospital yet suffer considerable morbidity and may have even higher long-term mortality than STEMI patients [3]. Even an emphasis on early invasive management in most NSTE-ACS patients continues to carry uncertainty regarding consistent benefit [19]. These clinical realities underscore the importance of understanding this syndrome at a more fundamental level in order to identify substrates for poor outcomes in these patients and to discover targets to improve evaluation and treatment pathways.

In order to understand NSTE-ACS fully, we first addressed the need for an adequate animal model to enable studies corroborating *in vivo* findings with *ex vivo* ultrastructural and functional assays. Using CMR techniques that readily translate to clinical application, we demonstrated myocardial T2 increase without significant myocardial necrosis by LGE. This paralleled mild TnI elevation but without ST elevation, plus ultrastructural and genetic changes that together corresponded to altered yet potentially viable myocardium. Our goal was not to produce a fixed amount of injury but rather to produce ischemic injury consistently without significant necrosis. The microsphere data demonstrated that reliable reductions in blood flow were achieved in the affected myocardium vs. the remote region in this dog model, despite the known presence of highly variable coronary artery collaterals in canines [20].

The translational value of these findings is evident when considering that the protocol can be readily implemented not only in prospective patient studies but also in preclinical studies aimed at better understanding the mechanisms that alter cellular and organ function. Our demonstration of altered expression of *CaMKIID* and associated increases in mitochondrial biogenesis, consistent with myocardial adaptation to acute ischemia [15,16], is an important first step in this direction as this protective pathway can be targeted through pharmacologic and non-pharmacologic mechanisms [21,22]. As such, CMR T2 alterations in the context of NSTE-ACS may identify patients who would benefit from early revascularization or other more novel approaches.

In finding a smaller but consistently positive difference between T2 in the lateral vs. anterior LV myocardium in sham animals, we further demonstrated physiological heterogeneity in T2 and mitochondrial size that may reflect previously-described regional variation in myocardial oxygen consumption [23]. Notably, human studies have also demonstrated variation in myocardial T2 across LV segments [24,25]. Given that mitochondria i) represent a large fraction of total myocardial volume, ii) are fluid enriched relative to muscle and iii) are much larger in the lateral wall, we suspect that regional differences in mitochondrial size could account for the observed variation in T2 values in the control group. Nonetheless, the T2 difference was significantly greater in the NSTE-ACS model vs. that seen in the sham animals.

A common explanation for increased T2 is the presence of myocardial edema. Other animal studies have found increased water content in ischemic myocardium correlated to the degree of stunning [26]. However, many of these studies involve more STEMI-like models with complete occlusion or multiple short cycles of brief occlusion causing cardioprotective post-ischemic conditioning. Our model, which lacks complete coronary occlusion and may better mimic human NSTE-ACS, did not produce a significant difference in wet vs. dry weight of affected myocardium. Elevated T2 in the absence of net water influx suggests an increase in the ratio of free-to-bound water in the affected myocardium as is known to occur as a consequence of the molecular and cellular responses to hypoxia, including acidosis [27-30]. However, the precise mechanisms responsible for myocardial T2 increase in ischemia remain poorly defined.

One potential mechanism involves mitochondria. Tissue aerobic respiration in the model group was significantly lower than in the sham dogs, indicating reduced mitochondrial oxygen consumption in the affected myocardium. Considering that mitochondrial ultrastructure and ADP-dependent respiratory capacity (*data not shown*) remained intact in the affected tissue during NSTE-ACS, the observed suppression of oxygen consumption likely reflects the shift from aerobic to glycolytic ATP production that characterizes myocardial stunning in the context of ischemia, which is regulated by HIF-1α and CaMKII in response to changes in tissue oxygen [31] and calcium [32], respectively. The observed change in *CaMKIID* expression and lack of *HIF-1α* response suggests that changes in intracellular calcium flux may contribute to the observed metabolic adaptations in the ischemic tissue. Moreover, high-level evidence indicates that sustained CaMKII activation can lead to myocardial remodeling and dysfunction [33]. Further investigation will be required to understand to what extent the cell is responding mal-adaptively or adaptively to ischemia and to delineate the time course for irreversible adaptive changes.

At the same time, we also demonstrated that significant ischemic cell death does not appear to be present in this model of NSTE-ACS. A large body of literature has documented that mitochondrial swelling is the earliest sign of impending necrotic cell death, and widespread high-amplitude mitochondrial degeneration portends irreversible ischemic injury [34,35]. In the present study, we specifically looked for the earliest signs of mitochondrial damage under electron microscopy along with contraction band necrosis by H&E staining. While EM studies were conclusively negative for necrosis and did not indicate mitochondrial damage, some H&E sections in both sham and model animals did show contraction band-like patterns. The interpretation of these patterns as an artifact of tissue preparation is further supported by the absence of enhancement on pre-mortem LGE images of the affected region and lack of abnormality on post-mortem TTC staining.

### Limitations

Despite the advantages of this large-animal model (e.g., compared to rodents [36]) in terms of modeling human ischemic heart disease, we recognize certain model limitations. First, we did not extend the period of ischemia beyond a 3-4 hour window; as such, the consequence of the observed T2 alterations in terms of longer-term tissue viability remains unclear. Due to concerns regarding adequate myocardial tissue availability after histopathological and oxygen respiration analyses, and the lack of invasive catheterization in unoperated shams, microspheres were only delivered in 5 animals. While we did not directly measure coronary artery stenosis or absolute coronary blood flow rates (only relative flow rates were obtained), this model produces mechanical restriction of the cross sectional coronary diameter (as dictated by the surgical protocol) and was shown to reliably reduce tissue perfusion, particularly following demand pacing, as reflected by tissue microsphere quantification. Absolute flow measurements may be incorporated into future studies. In terms of tissue bioenergetics, while it would be optimal to measure *in vivo* myocardial VO_2_ and ATP levels, the demonstrated decrease in tissue VO_2_ measured *ex vivo* under conditions of excess oxygen supply and associated changes in *CaMKIID* and mitochondrial biogenesis are in keeping with established bioenergetics adaptations to tissue hypoxia [15,37]. It is postulated that a lower metabolic state (stunning or hibernation) and a transition from aerobic towards anaerobic respiration is an adaptation that prevents lethal ATP depletion in the context of limited oxygen supply; further investigation using techniques such as phosphorus spectroscopy would help corroborate or refute this proposition.

## Conclusions

In a large animal model based on demand myocardial ischemia seen in the predominant form of NSTE-ACS in humans, myocardial T2 increases with corresponding, presumably adaptive, reduction in myocardial tissue respiratory function and preserved ultra structural findings indicating myocardial viability. The observed changes in oxygen metabolism and mitochondrial biogenesis, hallmarks of myocardial hibernation, suggest that antecedent T2 elevation without LGE identifies myocardium at risk of subsequent irreversible injury. Additional studies using this model to examine long-term changes and the impact of therapeutic maneuvers to restore normal T2 and mitochondrial function may further elucidate the syndrome of NSTE-ACS and guide management changes to improve outcomes.

## Abbreviations

ECG: Electrocardiography; ACS: Acute coronary syndrome; STEMI: ST-segment elevation myocardial infarction; NSTE-ACS: Non ST-segment elevation acute coronary syndrome; CMR: Cardiovascular magnetic resonance; LGE: Late gadolinium enhancement; TnI: Troponin I; ANOVA: Analysis of variance; H&E: Hematoxylin and eosin; TTC: Triphenyltetrazolium chloride.

## Competing interests

SVR and OPS receive research support from Siemens, who neither had input nor participation in any aspect of this work.

## Authors’ contributions

SVR and EDC were co-principal investigators of the study, coordinating all aspects of study design, study implementation, data analysis, and manuscript preparation. HC had primary responsibility for execution of all experimental procedures, data analysis, and manuscript preparation. GEB, the primary surgeon for this project, and RLH brought a wealth of veterinary experience and were closely involved with model development. EDC and MWJ performed the tissue analyses, including cell respiration assays, microscopy preparation, and TTC staining. GS performed PCR assays and data analysis and PBB aided with microscopy analysis. OPS contributed to MRI experiments, study design and data interpretation. TT assisted with image acquisition and blood analysis. GA was intimately involved with model development throughout and contributed to manuscript preparation. All authors read and approved the final manuscript.

## Authors’ information

Elliott D Crouser and Subha V Raman are co-senior authors.

## Supplementary Material

Additional file 1 **Additional methods and results for CMR and tissue assays. Figure S1.** Representative left ventricular myocardial tissue slices, post-TTC staining, demonstrate no evidence of necrosis. For each one, both sides of the slice are shown (left-right). The top three pairs (A-B, C-D, E-F) correspond to the affected regions (demonstrating the elevated T2 signal anatomically associated to the area with the papillary muscle, arrow) in 3 animals from the NSTE-ACS model group. Pair G-H corresponds to the affected region in a representative sham animal. Pair I-J corresponds to the remote region in a representative animal from the NSTE-ACS model group (the same one presented in pair A-B). Evidence of necrosis is absent in all slices, particularly within the affected regions. **Table S1.** CMR Scan Parameters. **Table S2.** Canine Primer Sequences Used for Real-time PCR. **Table S3.** Tissue Percent Water Content in the Myocardial Affected Region Alone and Normalized to That in the Corresponding Myocardial Remote Region. Click here for file
