# Supplementary material for: At-risk but viable myocardium in a large animal model of non ST-segment elevation acute coronary syndrome: cardiovascular magnetic resonance with ex vivo validation
Source: J Cardiovasc Magn Reson. 2013 Oct 9;15(1):94. doi: 10.1186/1532-429X-15-94 (PMC3852225; doi:10.1186/1532-429X-15-94)
Supplement: Additional file 1 — Additional methods and results for CMR and tissue assays. Figure S1. Representative left ventricular myocardial tissue slices, post-TTC staining, demonstrate no evidence of necrosis. For each one, both sides of the slice are shown (left-right). The top three pairs (A-B, C-D, E-F) correspond to the affected regions (demonstrating the elevated T2 signal anatomically associated to the area with the papillary muscle, arrow) in 3 animals from the NSTE-ACS model group. Pair G-H corresponds to the affected region in a representative sham animal. Pair I-J corresponds to the remote region in a representative animal from the NSTE-ACS model group (the same one presented in pair A-B). Evidence of necrosis is absent in all slices, particularly within the affected regions. Table S1. CMR Scan Parameters. Table S2. Canine Primer Sequences Used for Real-time PCR. Table S3. Tissue Percent Water Content in the Myocardial Affected Region Alone and Normalized to That in the Corresponding Myocardial Remote Region. [file 1532-429X-15-94-S1.pdf]

At-Risk But Viable Myocardium in a Large Animal Model of Non ST-Segment Elevation Acute Coronary Syndrome – SUPPLEMENTAL MATERIAL

Methods

Reagents: Unless otherwise stated, all chemicals were obtained from Sigma-Aldrich Corp. (St. Louis, MO) using the best available grade.

CMR Protocol: In all animals, NSTE-ACS model and sham, multiplane cine, T2 mapping, perfusion and late gadolinium enhancement (LGE) images were acquired using the scan parameters shown in **Table S1**. Breath-held, segmented, balanced, steady-stage free precession (SSFP) cine imaging[S1] (20-25 frames per cardiac cycle) was performed in a 3-chamber plane and contiguous short-axis planes to assess LV systolic function. Myocardial edema imaging was performed using a T2-prepared single-shot SSFP sequence with breath-holding to obtain T2-weighted images at three T2 preparation times (0, 24 and 55 ms)[S2]. After motion correction and pixel registration, the signal intensities in the 3 images at each pixel location were fitted to a T2 decay curve to compute the T2 at each pixel[S3]. T2 maps were acquired in 3-chamber and short-axis planes. First-pass perfusion imaging to visualize microcirculatory myocardial blood flow was acquired in 3 short-axis planes using a hybrid gradient echo-echo planar (GRE-EPI) technique during peripheral IV injection of 0.2 mmol/kg gadolinium-based contrast agent. At least 10 minutes after contrast injection, LGE imaging was performed in 3-chamber view and contiguous short-axis planes using an ECG-segmented, breath-hold fast gradient echo inversion-recovery prepped technique with appropriate inversion time selection to null normal myocardium.

Tissue Harvesting: Upon confirmed euthanasia, the heart was immediately removed and the left ventricle harvested. The regions corresponding to the affected and remote myocardia, as

identified by CMR (using the papillary muscles as anatomical landmarks), were carefully isolated with tissue samples segregated for blood flow determinations and the remainder immersed in ice-cold, air-saturated (bubbled with 5% CO_2) Krebs buffer (118 mM NaCl, 4.7 mM KCl, 1.5 mM $CaCl_2$, 25 mM $NaHCO_3$, 1.2 mM KH_2PO_4, 1.1 $MgSO_4$, 5.6 mM glucose) buffered with 10 mM HEPES (pH 7.4). Corresponding tissue samples from each myocardial region were then obtained for immediate tissue respiration, wet-to-dry weight and tissue necrosis [using 2,3,5-triphenyltetrazolium chloride (TTC)] analyses on fresh tissue. Additional samples were acquired and either fixed or frozen (-80°C) for later light and electron microscopy or gene (qRT-PCR) expression analyses (see below) respectively.

Tissues samples (~1 g), identified for wet-to-dry weight determination, were blotted dry and carefully weighed (W). After being in a drying oven for 72 hours at 75°C, samples were re-weighed (D) and the tissue water content (%) determined using the formula: (W – D/W)*100. Values were compared statistically between regions and across groups.

The presence or absence of necrosis within the affected and remote regions was examined by TTC staining. Corresponding tissue samples were sliced (2-3 mm thick) and momentarily rinsed with cold isosmotic PBS. Tissue slices were then immersed in a glass Petri dish containing a shallow layer of 2% TTC in PBS. The dish was covered with aluminum foil and incubated at 37°C for 30 minutes. The TTC solution was then replaced with 10% phosphate-buffered formalin and fixed overnight at room temperature. Stained sections were photographed on both sides within 24 hours. Normal tissue stained a deep red color with necrotic areas, if present, appearing unstained[S4].

Light and Electron Microscopy: Tissue samples (~0.5-1.0 g) obtained from the affected and remote myocardia designated for light microscopy were immersion fixed in 10% phosphate-buffered formalin for 72 hours. They were then routinely processed according to standard techniques for slide preparation wherein samples were blocked in paraffin, cut at 4 μm, de-paraffinized, and then stained with haematoxylin and eosin. Tissue histology was graded blindly for evidence of structural and/or cellular injury/abnormalities by pathologist review.

Additionally obtained samples (~0.5-1.0 g) from each myocardial region were processed for ultrastructural (electron microscopy) evaluation, as described elsewhere[S5,S6]. Briefly, the samples were immediately diced and submerged in a buffered isotonic fixative comprised of 4% paraformaldehyde, 2.5% glutaraldehyde and 0.1 M sucrose in 0.1 M phosphate buffer (pH = 7.4) at 4°C overnight. The next day, fixation was allowed to continue for approximately 2 hours at room temperature. Tissue pieces were then rinsed repeatedly in isotonic media containing 0.1 M sucrose in 0.1 M phosphate buffer (pH = 7.4), and post-fixed for 1 hour at room temperature in the same media containing 1% osmium tetroxide. After being rinsed again in the absence of osmium tetroxide, they were stored overnight at 4°C. The following morning, the fixed pieces were passively warmed to room temperature and dehydrated by passage through an ascending series of ethanol solutions. After rinsing with propylene oxide, they were infiltrated with and embedded in Spurr media that polymerized overnight at 60°C. The next day, thin sections (80-90 nm) were cut on a Reichart Ultracut E microtome, mounted on copper grids, and stained with 2% uranyl acetate and Reynolds lead citrate. The stained grids were examined using a FEI Tecnai G2 Spirit transmission electron microscope operated at 80 kV.

High resolution digital images (magnification: 11,000X) of myocardial tissue mitochondria were acquired at 2 randomly selected and separated areas within each region on

each of 2 different prepared grids. The mitochondria were then quantitatively evaluated using Image J analysis software. The outlines of all mitochondria present in each image (~8-10 per digital image) were manually traced along the edge of their outer membrane. Once traced, each individual mitochondrion was independently evaluated using digital parameters. Traditional evaluations of mitochondrial morphology focus mainly upon the size and shape of the mitochondria. Therefore, three digital parameters that related to these subjective scoring parameters were selected[S7]. The mitochondrial parameters comprised: area, aspect ratio (long axis/short axis), and perimeter. Measurements of these parameters were then made on each individual mitochondrion (i.e., ~600 mitochondria were individually investigated). Average parameter values were then determined for each digital image. Values obtained for the affected myocardial mitochondria were normalized to that averaged from the corresponding remote myocardial mitochondria for the sake of comparison between the groups.

Ex Vivo Myocardial Tissue Oxygen Consumption: To evaluate the effect of ischemia upon cellular respiration in our NSTE-ACS model, small myocardial sections (~0.2-0.3 grams) from the affected and remote regions were finely diced and mixed in a bath containing 4 mL of air (bubbled with 5% CO_2)-saturated Krebs solution (118 mM NaCl, 4.7 mM KCl, 1.5 mM $CaCl_2$, 25 mM $NaHCO_3$, 1.2 mM KH_2PO_4, 1.1 $MgSO_4$, 5.6 mM glucose) buffered with 10 mM HEPES (pH 7.4). The O_2 concentration in the bath was measured polarographically over time using a Clark-type electrode connected to an O_2 monitor (YSI model 5300, Yellow Springs Instrument Co., Inc., Yellow Springs, OH). O_2 consumption by the myocardial cells was calculated assuming an initial O_2 concentration of ~240 nmol/mL. Tissue mitochondrial

respiration was determined by subtracting non-mitochondrial oxygen consumption [i.e., after treatment with electron transport inhibitors rotenone (5 μM) and antimycin A (5 μM).

Mitochondrial respiration rates were calculated for several small myocardial sections from each region, then averaged to determine the mean tissue respiration rate (nmol O_2/mg protein/min) for that region. For each animal, the ratio of the respiration rate in the affected region to that in the remote region was determined and then compared between the model and sham groups.

Gene Expression as Determined by Real-time PCR: Frozen tissue samples from each of the myocardial regions obtained from each animal and stored at -80°C were bead homogenized in ice-cold TRIzol® reagent (Invitrogen Corp., Life Technologies™; Grand Island, NY) according to the manufacturer's protocol to isolate total RNA. Tissue samples were added to 1.0 mm Zirconia/Silica beads (10 % v/v) and 1 ml of cold TRIzol® reagent and then lysed using a Mini-Bead Beater cell disrupter at 5000 rpm for 10 sec, repeated four times at 1 min intervals while being stored on ice. Tissue homogenates were then subjected to high-speed centrifugation (10,000 x *g* for 5 min at 4°C) to pellet the beads and cellular debris.

Phase separation of the RNA in the resultant supernatant was carried out following the addition of 200 μl of chloroform, vigorous mixing for 15 seconds, incubation for 2-3 minutes at room temperature and centrifugation at 12,000 x *g* for 15 minutes at 4°C. The top RNA-containing phase was carefully transferred to a new tube and gently mixed with 500 μl of isopropanol to precipitate the RNA. After a 10-minute incubation at room temperature, the RNA was pelleted by centrifugation at 12,000 x *g* for 10 minutes at 4°C. After careful removal of the supernatant, 75% ethanol was added to wash the RNA pellet which was then vortexed and

centrifuged at 7500 x *g* for 5 min at 4°C. The ethanol was removed, and the pellet was allowed to air dry. The RNA pellet was then dissolved in 30 µl of RNase-free water by gentle pipetting, the RNA concentration determined, and then all samples stored at -80°C for later analyses.

Gene expression for select canine genes (**Table S2**) in both the affected and remote myocardial regions was determined using real-time PCR. Briefly, target RNA was reverse transcribed using the High Capacity cDNA Reverse Transcription (RT) kit (Invitrogen Corp., Life Technologies™) to generate extremely high-quality, single-stranded cDNA. In a 20 µl RT reaction, 2.5 µM random primer, 600 ng total RNA, 0.4 U/µl RNase inhibitor, 1.25 U/µl MultiScribe™ Reverse Transcriptase (Applied Biosystems, Life Technologies™), and 500 µM dNTP were mixed in the presence of 50 mM Tris-HCl (pH 8.3), 75 mM KCl, 3 mM $MgCl_2$ and incubated at 25°C for 10 minutes, 37°C for 120 minutes, and then 85°C for 5 seconds. For each experiment, RT-minus controls (i.e., RNA samples that were treated similarly but without the addition of MultiScribe™ RT enzyme) were included to provide negative controls for subsequent PCR reactions. Real-time PCR was then performed in a 20 µl mixture containing 5 µl diluted cDNA sample (1:50), 4 µl of a 2.5 µM primer mix (500 nM final concentration of each primer pair) (**Table S2**) and 10 µl of 2×Power SYBR® Green PCR master mix (Applied Biosystems, Life Technologies™). All of the primers spanned an intron, ensuring that gDNA contamination was not present. Primer pairs were validated by real-time PCR and high-resolution gel electrophoresis to have a single band of desired size that was free of primer dimers. Amplification and detection were performed in duplicate and achieved with the 7500 Fast Real-Time PCR System (Applied Biosystems, Life Technologies™) using the profile of 50°C for 2 min, 95°C for 10 min, and 40 cycles of 94°C for 15 sec and 60°C for 1 min. Relative

copy numbers and expression ratios for all genes of interest were normalized to the expression of the housekeeping gene, *β-actin*.

References

S1. Bundy J, Simonetti O, Laub G, Finn JP. Segmented TrueFISP imaging of the heart. *Proceedings of the 7th Annual Meeting of the International Society for Magnetic Resonance in Medicine.* 1999:P1282.

S2. Giri S, Chung YC, Merchant A, Mihai G, Rajagopalan S, Raman S V, Simonetti OP. T2 quantification for improved detection of myocardial edema. *J Cardiovasc Magn Reson* 2009; 11:56.

S3. Giri S, Shah S, Xue H, Chung YC, Pennell ML, Guehring J, Zuehlsdorff S, Raman S V, Simonetti OP. Myocardial T(2) mapping with respiratory navigator and automatic nonrigid motion correction. *Magn Reson Med* 2012; 68:1570–1578.

S4. Isayama K, Pitts LH, Nishimura MC. Evaluation of 2,3,5-triphenyltetrazolium chloride staining to delineate rat brain infarcts. *Stroke* 1991; 22:1394-1398.

S5. Crouser ED, Julian MW, Dorinsky PM. Ileal VO_2-DO_2 alterations induced by endotoxin correlate with severity of mitochondrial injury. *Am J Respir Crit Care Med* 1999; 160:1347-1353.

S6. Joshi MS, Julian MW, Huff JE, Bauer JA, Xia Y, Crouser ED. Calcineurin regulates myocardial function during acute endotoxemia. *Am J Respir Crit Care Med* 2006; 173:999-1007.

S7. Joshi MS, Crouser ED, Julian MW, Schanbacher BL, Bauer JA. Digital imaging analysis for the study of endotoxin-induced mitochondrial ultrastructure injury. *Anal Cell Pathol* 2000; 21:41-48.

Figure Legends

Figure S1: Representative left ventricular myocardial tissue slices, post-TTC staining, demonstrate no evidence of necrosis. For each one, both sides of the slice are shown (left-right). The top three pairs (A-B, C-D, E-F) correspond to the affected regions (demonstrating the elevated T2 signal anatomically associated to the area with the papillary muscle, arrow) in 3 animals from the NSTE-ACS model group. Pair G-H corresponds to the affected region in a representative sham animal. Pair I-J corresponds to the remote region in a representative animal from the NSTE-ACS model group (the same one presented in pair A-B). Evidence of necrosis is absent in all slices, particularly within the affected regions.

Figure S1

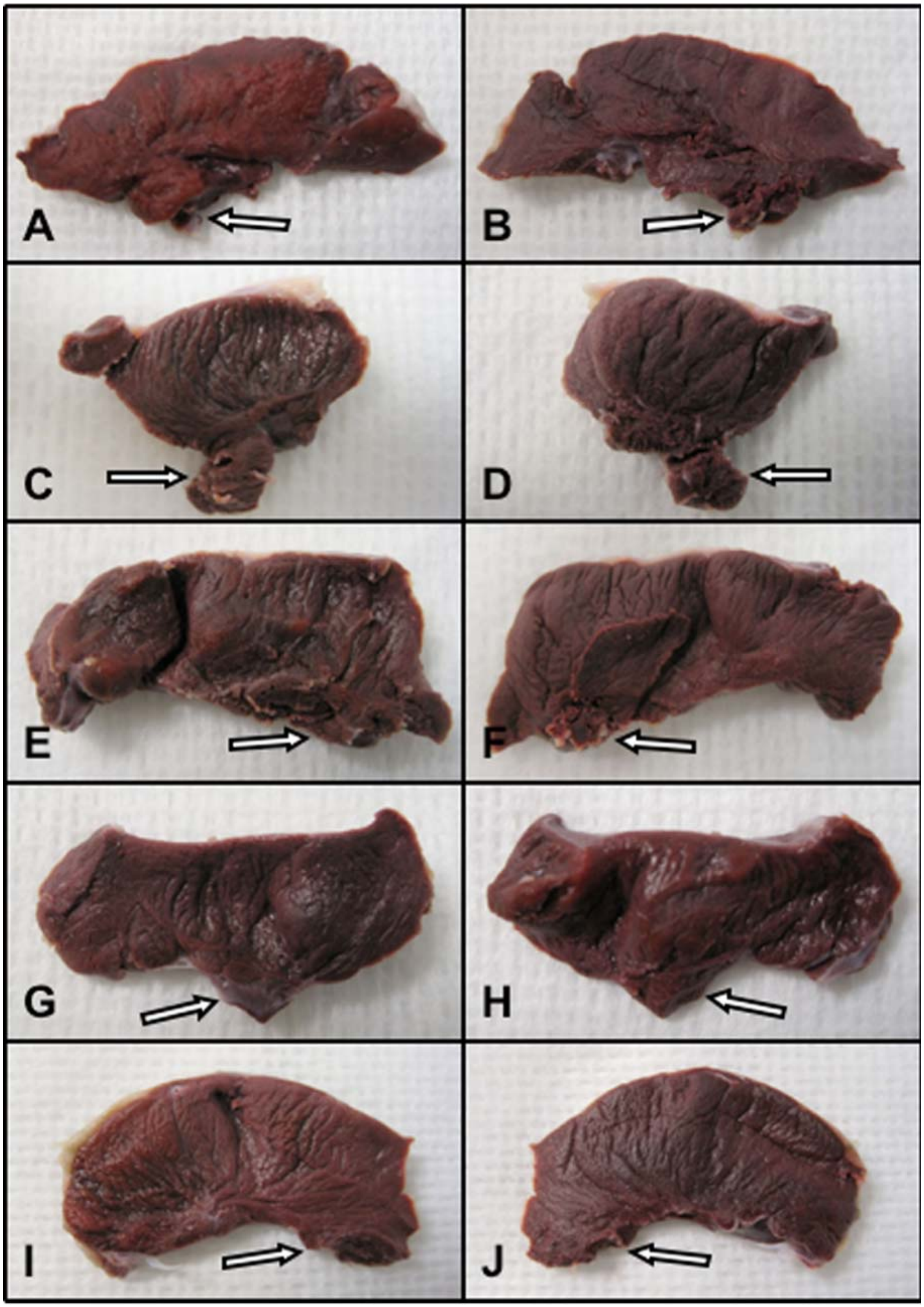

Table S1: CMR Scan Parameters.

	SSFP cine	T2 Mapping	Tagging	Perfusion	LGE
**Repetition time**	3 ms	3×(R-R interval)	40 ms	132 ms	R-R interval
**Echo time**	1.3 ms		3.0 ms	1.0 ms	4.2 ms
**Bandwidth**	930 Hz/pixel	1445 Hz/pixel	260 Hz/pixel	1953 Hz/pixel	130 Hz/pixel
**Flip angle, deg**	65	40	15	25	25
**In-plane spatial resolution, mm**	1.5 × 1.5	2.2 × 2.8	8 mm tag spacing	2.8 × 3.4	2.2 × 2.0

SSFP = steady-state free precession; LGE = late gadolinium enhancement

Table S2: Canine Primer Sequences Used for Real-time PCR.

Gene	Amplicon (bp)	Primer Sequence	
ATP Synthase 6	148	Sense	5'-GGACGAACCTGAGCTCTCATA-3'
		Antisense	5'-TTACTGTCCCTGCTCATAGGG-3'
CaMKIID	128	Sense	5'-GTGGAATGCCAAAGACAATG-3'
		Antisense	5'-TGGAATACAGGATGGCTTGA-3'
COX I	134	Sense	5'-TCTGACTCCTTCCTCCATCC-3'
		Antisense	5'-TGTAAGGTCAACGGATGCTC-3'
COX II	137	Sense	5'-GCCACCAATGATACTGAAGC-3'
		Antisense	5'-TATTGGGAGGACAACTCGGT-3'
COX III	191	Sense	5'-TCCTACCGGCATTATTCCTC-3'
		Antisense	5'-ATTCGGAGGCCTGTAATAGC-3'
COX IV	106	Sense	5'-GTACCGCATCAAGTTCAACG-3'
		Antisense	5'-GAGAGCAGTGAAGCCGATAA-3'
HIF-1α	193	Sense	5'-GACAGCTGACATCCAACCTG-3'
		Antisense	5'-AAGTCCACCTGGCTCTGTCT-3'
ND1	191	Sense	5'-AGGTCCTTTCGCCCTATTCT-3'
		Antisense	5'-GGAATCGAGGGTATGATGCT-3'
ND4	199	Sense	5'-CCCGATGAGGAAATCAAACT-3'
		Antisense	5'-ATGCATGCCAGTCATAGGAA-3'
ND6	109	Sense	5'-AAGAGGCTATGGGTATTGCG-3'
		Antisense	5'-CCACGGGTAACTTCCATGAT-3'
β-actin	152	Sense	5'-GGTCATCACTATTGGCAACG-3'
		Antisense	5'-TGTTGGCATAGAGGTCCTTG-3'

Table S3: Tissue Percent Water Content in the Myocardial Affected Region Alone and Normalized to That in the Corresponding Myocardial Remote Region.

Groups	Percent Water Content (Affected Region)	Wet-to-Dry Ratio (Normalized to Remote Region)
**Sham**	79.1 ± 0.5	1.003 ± 0.005
**NSTE-ACS Model**	77.9 ± 0.9	0.994 ± 0.002
**Unoperated Control**	79.1 ± 0.9	1.002 ± 0.002
**Surgery (All)**	78.5 ± 0.9	0.999 ± 0.007
